# Fourteen-year change in activities of daily living of a quadriplegic, ventilator-managed patient injured by high cervical spinal cord injury during infancy: a case report

**DOI:** 10.1186/s12887-022-03573-y

**Published:** 2022-08-25

**Authors:** Yoshinori Yasuoka, Yukihide Nishimura, Tokio Kinoshita, Yumi Koike, Yasunori Umemoto, Fumihiro Tajima

**Affiliations:** 1 Division of Rehabilitation, Wakayama Medical University Hospital, 811-1 Kimiidera, Wakayama, 641-8510 Japan; 2 Department of Rehabilitation Medicine, Wakayama Medical University, 811-1 Kimiidera, Wakayama, 641-8509 Japan; 3 Department of Rehabilitation Medicine, Iwate Medical University, 2-1-1 Idaidouri, Yahaba-cho, Shiwa-gun, Iwate 028-3694 Japan

**Keywords:** ADL, Disability, Child, Quality of Life, Functional Independence Measure, Electric Wheelchair

## Abstract

**Background:**

Rehabilitation of patients with high cervical spinal cord injury (CSCI) to improve activities of daily living (ADL) is challenging due to severe paralysis. In addition, pediatric patients with CSCI are rare, and literature describing ADL changes as the patient grows are limited. In this case report, we present the long-term change in ADL over time in a girl with severe high CSCI from an injury during infancy.

**Case presentation:**

A 2 years and 6 months old girl, who was injured in a traffic accident, was diagnosed with C3 CSCI, resulting in complete quadriplegia and respiratory paralysis below C3. Thus, she was managed with a ventilator. Rehabilitation for quadriplegia, respiratory dysfunction, and autonomic neuropathy was started on the fifth day after the injury while she was in the intensive care unit. Six months after the injury, the patient was transferred to a hospital. Thereafter, she was discharged with nursing and care guidance provided to her family and environmental changes at home. Afterwards, she continued to acquire skills through writing training using a mouse stick, computer operation training, and electric wheelchair operation training, which enabled her to improve her ADL despite her severe disability. In terms of education, she was able to go through a regular elementary school, a regular junior high school, and then to a senior high school of a support school.

**Conclusions:**

We believe that training that utilizes current technology and changes in the environment that are appropriate for daily life are important for improving the ADL of children with severe CSCI.

## Background

Spinal cord injury (SCI) in pediatric patients is rare, representing less than 10% of all cases [1]. Although training for activities of daily living (ADL) should naturally be based on the developmental level for children with SCI [2], literature on ADL changes with growth over time are limited. It has also been reported that cervical spinal cord injury (CSCI) in early childhood involves the C2-3 levels due to the greater range of motion of the higher cervical spine [3]. Patients with C2-3 CSCI may develop respiratory muscle paralysis and require a ventilator, making rehabilitation difficult. CSCI also results in a catastrophic loss of upper extremity function [4]. The degree of quadriplegia affects independence and quality of life (QOL) [5], and more severe disability can decrease life satisfaction [6]. In general, rehabilitation for patients with CSCI is based on the level of injury as defined by the International Standard for Neurological Classification of Spinal Cord Injury (ISNCSCI) [7]. Treatment sessions are planned and implemented with the goal of improving mobility and participation to the greatest extent possible, taking into account the remaining motor and sensory function while minimizing secondary complications [8, 9]. Independent activities are more limited at higher levels of injury. For example, patients with C8 CSCI can use a manual wheelchair and perform ADL independently, whereas patients with C5 CSCI require assistance in operating a manual wheelchair [10, 11]. In addition, all ADL require full assistance at higher injury levels. Patients with higher levels of CSCI require special environmental changes, such as a motorized wheelchair to gain mobility, and it is important that these changes be appropriate for each patient [12–14]. Environmental changes are not only limited to equipment but also include various aspects such as work or school environment and the understanding of the people involved [15]. This case report describes the changes in ADL and rehabilitation of a girl with severe high CSCI who was injured in early childhood.

## Case presentation

### Medical history

**Fig. 1:**
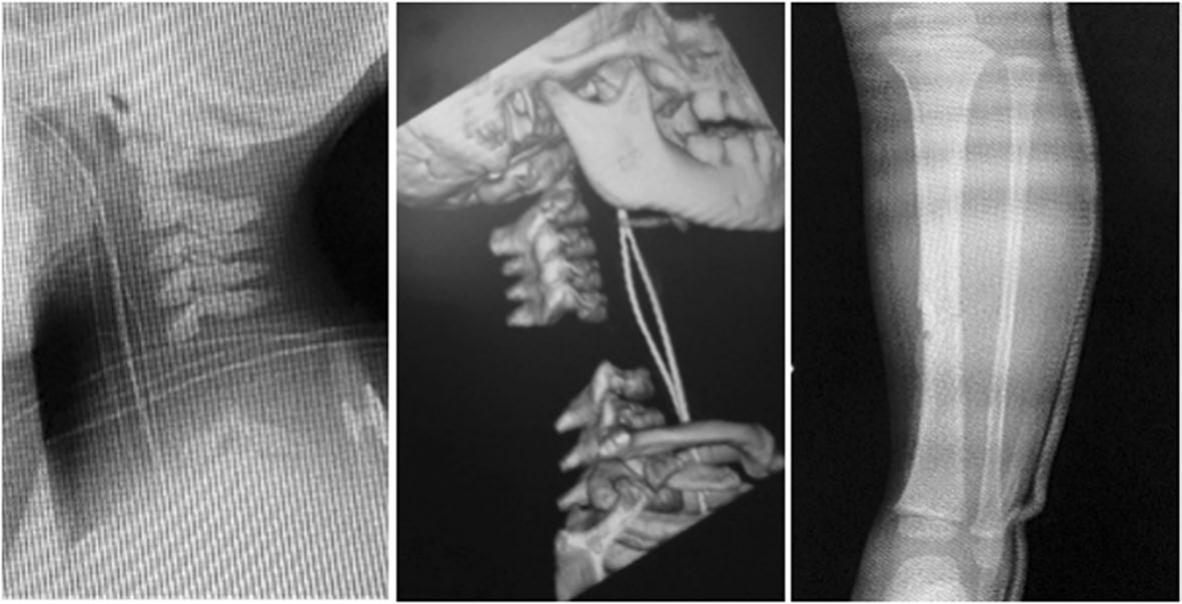
X-ray and CT images at the time of injury

**Fig. 2:**
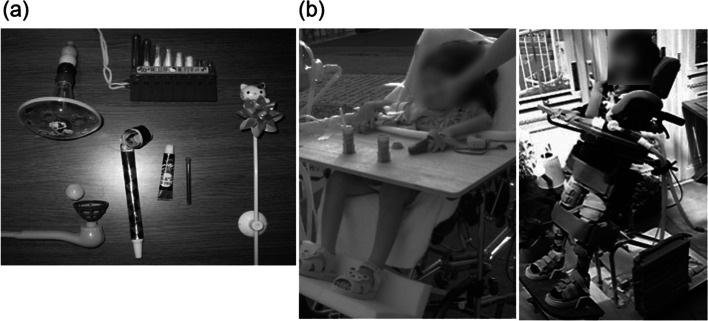
Toys used for respiratory rehabilitation (**a**) and rehabilitation training using the fabricated sit-to-stand device and standing table (**b**)

**Fig. 3:**
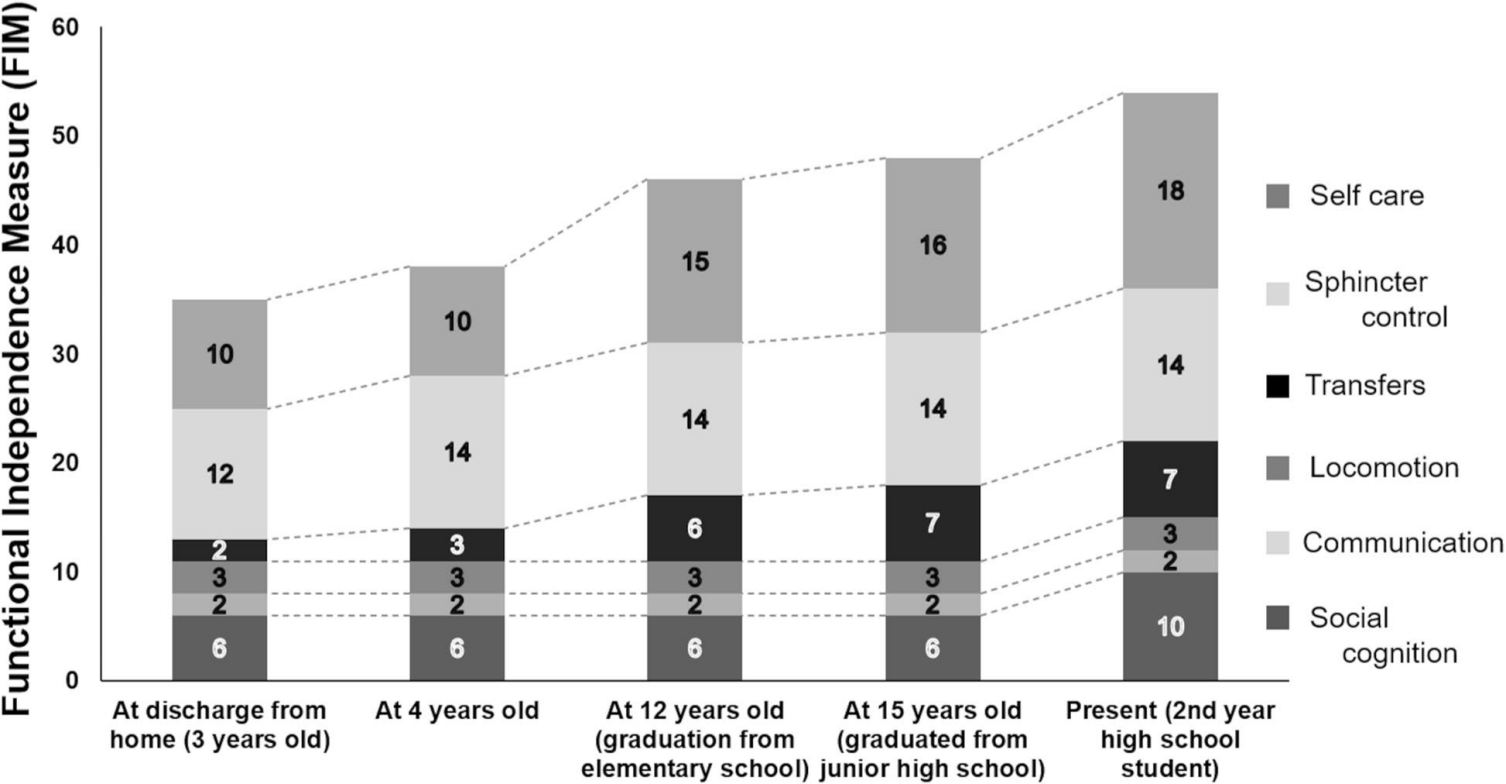
Changes in Functional Independence measure (FIM) over time

The patient was 2 years and 6 months old when she was injured in a traffic accident between a private car and a truck. She underwent cardiopulmonary arrest and was rushed to an acute care hospital where she was resuscitated. She was diagnosed with C3 CSCI and tibial fracture (Fig. 1). Acute physical findings revealed left conjugate deviation of the eyes, complete quadriplegia below C3, and respiratory paralysis. Thus, she was managed on a ventilator. Treatment consisted of posterior fusion and halo vest for C3 CSCI and fixed seine for her tibial fracture. Rehabilitation was started in the ICU on the fifth day after the injury. During the acute phase, rehabilitation focused on respiratory rehabilitation and early mobilization for complete quadriplegia below C3, respiratory dysfunction, and orthostatic hypotension. Training was conducted through play using toys such as soap bubbles and whistles to increase ventilation and, as appropriate, with the use of assistive devices such as sit-to-stand and standing tables. (Fig. 2). Her general condition gradually improved and she was discharged from the ICU 46 days after the injury. Six months after the injury, she was transferred to a local hospital for environmental adjustment for home discharge under ventilator control with ADL requiring full assistance. The American Spinal Injury Association (ASIA) motor score was 0. Her ADL status at the time of discharge is shown in Fig. 3 using the Functional Independence measure (FIM). The total FIM score was 35 points, and all items such as selfcare and locomotion required full assistance.

The FIM is one of the most reliable and widely used methods in assessing the basic quality of ADLs for people with disabilities [16]. The FIM is designed to assess the amount of assistance needed for a person with a disability to safely and effectively perform basic ADLs and consists of 18 items. These activities include minimal skills related to self-care, sphincter control, transfer, locomotion, communication, and social cognition. Each item was rated on a 7-point scale and graded according to common criteria. Patients are classified as "7: Complete independence" if they are able to perform activities in a completely normal manner, and "6: Modified independence" if they need tools to assist with activities or need more time to perform them. The FIM is also graded according to the degree of assistance: "5: Supervision," "4: Minimal Assistance," "3: Moderate Assistance," "2: Maximal assistance," and "1: Total Assistance” [17]. In this case report, the same experienced physiotherapist in charge of the patient calculated the total, motor, and cognitive scores.

### Rehabilitation for school readiness

**Fig. 4:**
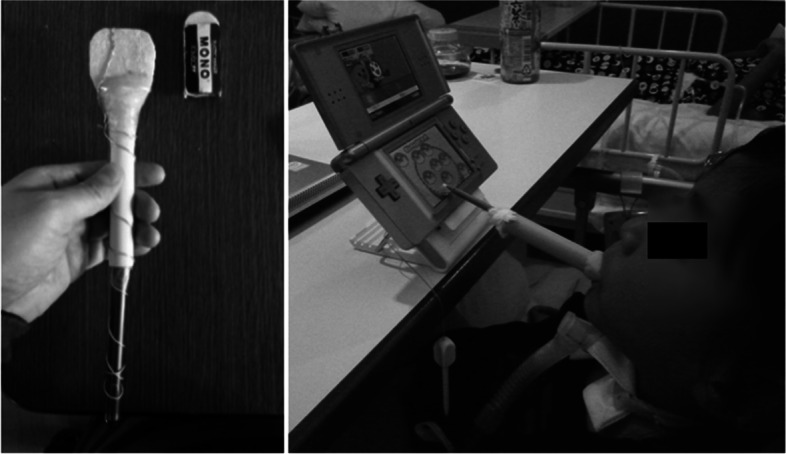
Mouse stick assembly and training scene

**Fig. 5:**
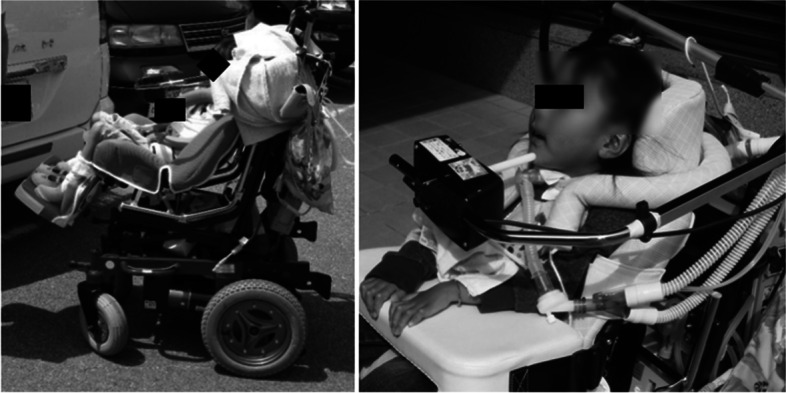
Electric wheelchair operated by chin control

Rehabilitation until school age was conducted for developmental and mobility impairments. Specific examples include the assembly of a mouse stick and an electric wheelchair. Figure 4 shows the actual mouse stick and the training scene. Using the mouse stick, the child underwent repeated training to write through games and to operate a computer until she entered school. Figure 5 shows the electric wheelchair. The wheelchair was made when she was 4 years and 6 months old. Since she still had quadriplegia, she was repeatedly trained to safely maneuver the wheelchair with chin control. As a result, at 6 years of age, she was able to go to the local elementary school like most children.

### Progress in schooling

**Fig. 6:**
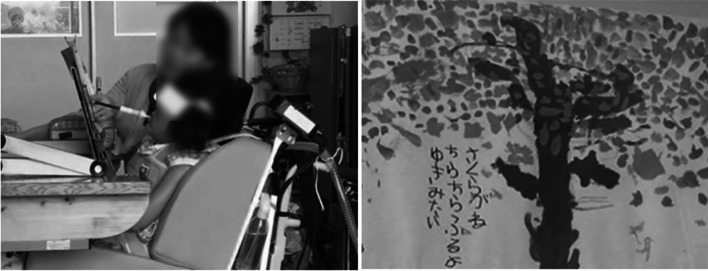
Patient attending a lecture using a mouse stick and the pictures and text she drew

As for her education, she entered a local regular elementary school at the age of 6 years, a regular junior high school at 12 years, and a support high school at 15 years. During her elementary school life, she was able to participate in field trips and sports events with her classmates using an electric wheelchair. She attended regular lectures as much as possible by using a mouse stick to write and operate a computer. In addition, lectures were held with one teacher accompanying the students to assist them depending on the content of the lecture. Figure 6 shows her actually attending the lecture using the mouse stick and the pictures and letters she drew. In addition, Fig. 3 shows her FIM score at the end of elementary school over time. The total FIM score increased to 46 points, with improvement in locomotion, communication, and social cognition. The improvement was attributed to the fact that she was able to operate a power wheelchair and move around while supervised. She was also able to interact with others at her own will. There was no change in the ASIA motor score.

**Fig. 7:**
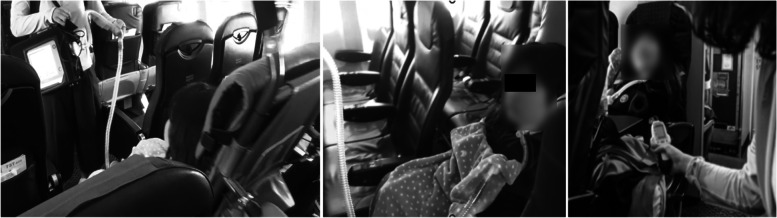
Boarding the plane and checking vitals

During her middle school years, she experienced travel by air. In her life up to junior high school, her only means of transportation was a motorized wheelchair and full-assistance private car transportation. Therefore, the first time she flew, a rehabilitation physician and a physical therapist boarded the plane with her to check her boarding and vitals (Fig. 7). This experience enabled the family to travel alone thereafter. As she grew older, her knowledge further improved, and she was able to use a computer to conduct research. This led to an improvement in the locomotion and social cognition items in the FIM at the end of middle school, with a total score of 48 (Fig. 3).

**Fig. 8:**
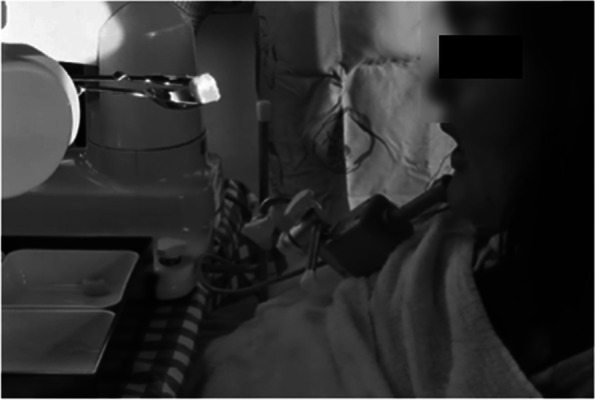
Eating motion with the use of the robot “My Spoon”

**Fig. 9:**
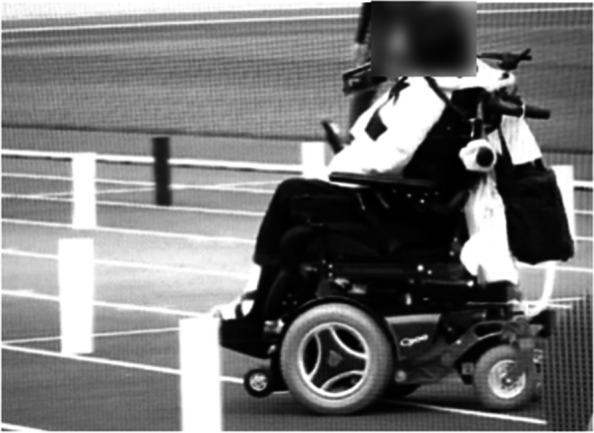
Participation in sports for the disabled

After entering a special support high school, rehabilitation using a robot was started to improve self-care skills. As a result, she was able to use the assistive robot to perform eating movements (Fig. 8). By placing food in a container and operating a chin controller, it became possible for the patient to eat what she wants to, eat in the order she wants to, and choose the timing of her feedings. In addition, her physical education lectures enabled her to participate and play in local sports tournaments (Fig. 9). As a result, her total FIM score has increased to 54 points in her second year of support school. Her self care and social cognition items have improved. There was no change in the ASIA motor score. Details of her current total FIM score are shown in Table 1.

**Table 1:** All domains of FIM score at present (2nd year of support school)

Category	Task Type	♯	Task	Score
Motor	Self-care	1	Eating	5
		2	Grooming	1
		3	Bathing	1
		4	Upper body dressing	1
		5	Lower body dressing	1
		6	Toileting	1
	Sphincter control	7	Bladder management	1
		8	Bowel management	1
	Transfers	9	Bed to chair transfer	1
		10	Toilet transfer	1
		11	Tub/shower transfer	1
	Locomotion	12	Walk/wheelchair	6
		13	Stairs	1
Cognitive	Communication	14	Comprehension	7
		15	Expression	7
	Social cognition	16	Social interaction	7
		17	Problem solving	4
		18	Memory	7

**Fig. 10:**
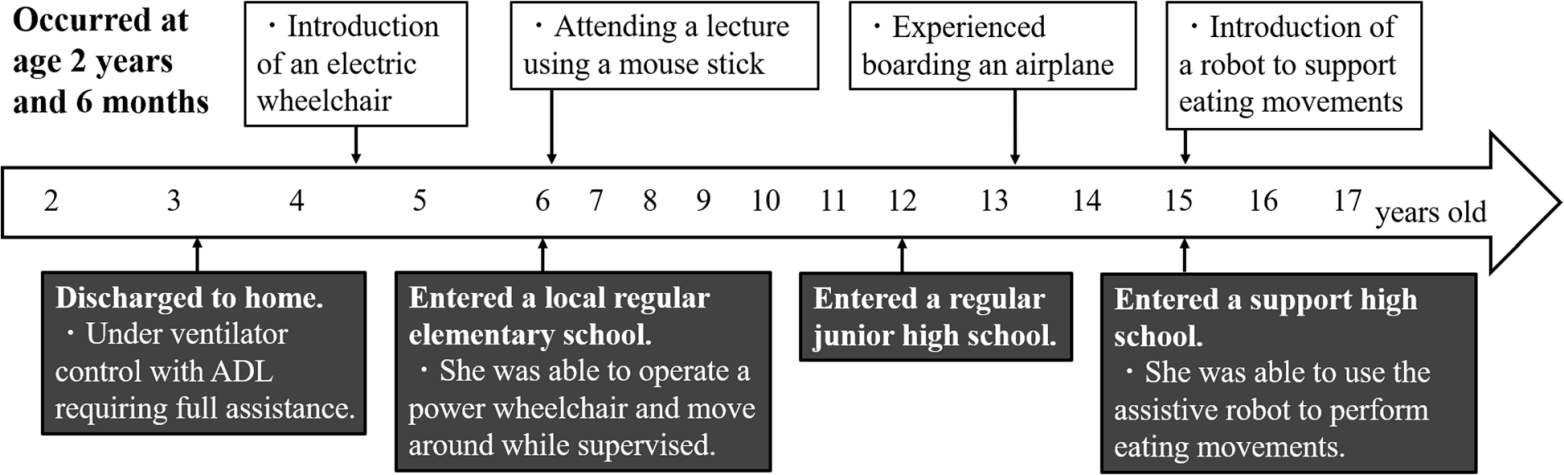
Summary of her progress shown in chronological order

In addition, the chronological progression of this long period of time is summarized in Fig. 10.

### Medical progress

**Fig. 11:**
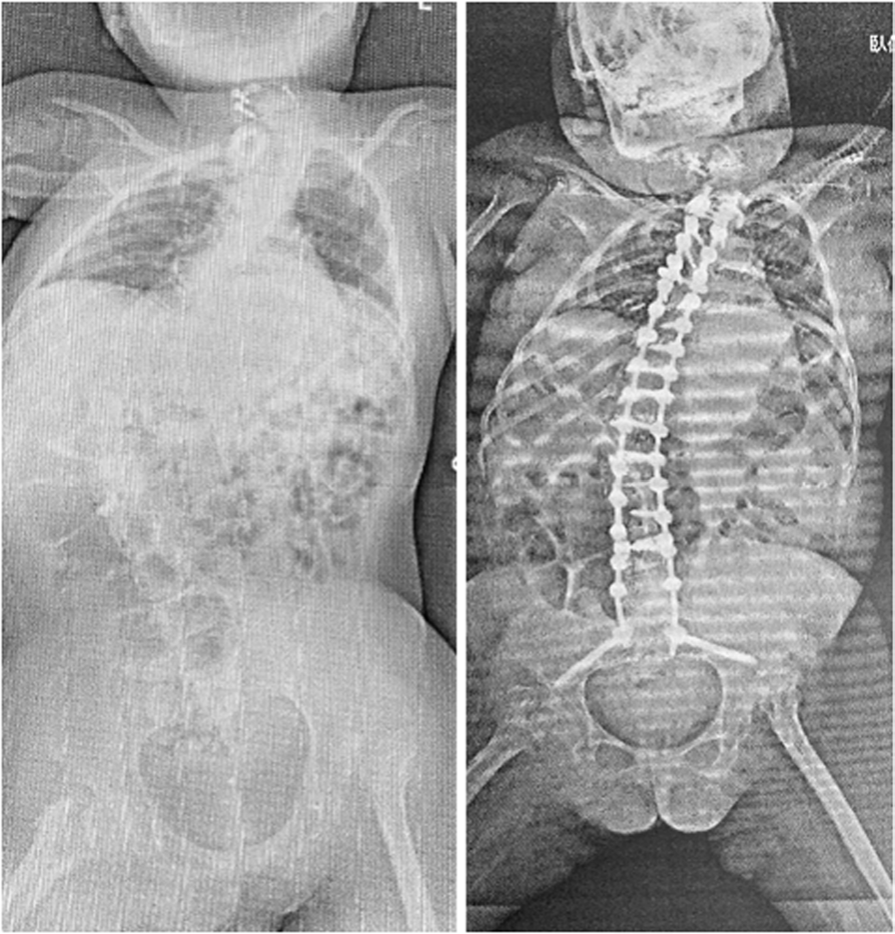
Pre- and postoperative X-rays of posterior spinal instrumentation and fusion (PSIF) for scoliosis

She had quadriplegia and respiratory paralysis as sequelae of her CSCI. In addition, her ASIA score did not change over time, and weaning from the ventilator was difficult. The ventilator mode was controlled mechanical ventilation (CMV) at the time of injury, and was changed to synchronized intermittent mandatory ventilation (SIMV) as her level of consciousness improved and her respiratory status changed. After discharge from the ICU and transfer to the general pediatric ward, the ventilator setting was gradually changed from SIMV to continuous positive airway pressure (CPAP) mode, and she was discharged home on a portable ventilator. During the following period until schooling, she felt better with the respiratory frequency (f) set at 3 breaths rather than 0. During schooling, the ventilator was set to pressure support (PS); 12 mmH_2_O, positive end-expiratory pressure (PEEP); 4 mmH_2_O, f; 3 times (6 times at bedtime) and has remained at the same settings to date. Furthermore, during the course of the disease, paralysis and spasticity resulted in spasticity of the lower extremities and scoliosis of the spine. Physical therapy, orthotics, and botox injections were administered as needed for the lower limb. Functional scoliosis was detected when she was 8 years old, and a trunk brace was prescribed. By 12 years of age, her scoliosis progressed to a Cobb angle of 43° in T7-L3 and 45 degrees in C5-T7. By the age of 13 years and 10 months, the Cobb angle progressed to 70° in T6-L4 and 49° in T6-C5. This resulted in difficulty sitting and lying on the left side; hence, a posterior spinal instrumentation and fusion of Th2-S2 (PSIF) was performed (Fig. 11). Postoperatively, pleural effusion and pneumonia were observed. With appropriate medical treatment and rehabilitation, the patient was discharged 14 days after surgery with the same respiratory setup as preoperatively.

### Informed consent

Data collection was carried out according to the international ethical standards with humans of the Declaration of Helsinki. Written informed consent was obtained from the patient and her family to publish any potentially identifiable images or data included in this article. This study conforms to all case report guidelines and reports the required information accordingly.

## Discussion and conclusions

This case report describes the rehabilitation of a child with severe high CSCI sustained in infancy. Despite the fact that the child required a ventilator and was severely disabled with C3 complete quadriplegia, she was able to improve her ADL by continuing rehabilitation as she grew. We also believe that this improvement in ADL capacity has improved the QOL of the patient and her family. This case report is the first long-term demonstration that appropriate rehabilitation techniques using the latest technology can improve the QOL of patients with high CSCI.

Her motor paralysis and respiratory function did not change with age after the injury. However, the FIM results showed an increase of 8 points in locomotion and social cognition from age 4 to 12 years when she graduated from elementary school. This may have been mainly caused by the introduction of an electric wheelchair at the age of 4 years and 6 months. A previous study reported that the early introduction of a wheelchair and self-mobility for children with SCI had a positive impact on the children's ability to learn, participate with peers, increase self-confidence, and reduce depression [18–20]. Normally, 4-year-olds grow up walking on their own feet and exploring their surroundings [13]. She was able to maneuver her electric wheelchair, which enabled her to move herself to the person she wanted to talk to and interact with others. We believe that this has had a positive effect on her mental growth and learning ability. In addition, we believe that the assembly of a mouse stick, which enabled her to write and operate a computer, also contributed significantly to the improvement of her ADL. Even for children without physical disabilities, early mastery of reading and writing affects all other facets. Thus, supporting reading and writing has been one of the most important issues to be considered [21, 22]. She was able to draw and write using a mouse stick, and furthermore, she was able to compensate for her writing speed by becoming more proficient at operating a computer. As a result, she was able to enroll in the regular schools that most children attend in elementary and junior high school.

The FIM scores improved in the locomotion and social cognition items at the end of middle school. Specifically, the scores increased in wheelchair mobility and problem solving. However, no new equipment was introduced during her middle school life. The factors that contributed to the increase in FIM scores were her daily learning and experience. These enabled her to understand traffic rules and behave safely according to her surroundings. Her ability to gather information also improved using a computer. In addition, it is important to note that her experience in the airplane boarding process has enabled her to use public transportation. She and her family were able to travel without medical support after experiencing the plane boarding process. A review by Lindsay et al. concluded that being able to use public transportation and travel independently is an important factor in maintaining quality of life and community participation [23]. In addition, a study on the use of public transportation among people with cerebrovascular disorders reported that a lack of confidence a factor that prevents people with physical disabilities from using public transportation [24]. One way to remove this barrier is to provide patients with successful experiences [25]. The first time that the patient boarded the plane, she was accompanied by a physiatrist and a physical therapist. We believe that this kind of involvement in real-life situations contributed to the improvement of her ADL. Although the improvement in the total FIM score was only 2 points higher than when she graduated from elementary school, the QOL of the patient and her family was considered to have improved significantly.

The improvement in her FIM score after entering a support school can be attributed to the introduction of a robot [26] that assisted her in eating. She was also able to engage in sports under the disability-oriented educational environment of the support school. Previous studies have shown that involvement in sports brings about social interaction, contributing to psychological benefits and life satisfaction [27–29]. In addition, the benefits of meal-assist robots include the ability to enjoy meals with family members who used to be caregivers and the elimination of reservation felt by patients toward caregivers by being able to perform meal movements by themselves [30]. Consistent with these reports, we believe that the improvement in her ADL improved her QOL.

With regard to the course of the sequelae of her CSCI, her neurological deficits did not improve as she grew older and her ASIA score did not change. She developed quadriplegia at a young age and was forced to lead a sedentary life because of her immobility. Her medical history was consistent with the expected outcome [31] for a child injured at less than 5 years of age. The review indicated, for example, that 96% of patients would develop scoliosis and 61% would experience spasticity.

In this case, rehabilitation using the current technology enabled improvement in ADL over time, even if improvements in physical functions did not occur. Through our experience with her rehabilitation, we have realized the importance of national and local government support for people with disabilities. For example, the patient was given an electric wheelchair at the age of 4 years and 6 months. However, there is no support for the purchase of electric wheelchairs for 4-year-old children in Japan. Furthermore, disability is strongly associated with poverty globally. In resource-poor settings, most people who need a wheelchair cannot afford to buy one on their own [32]. We hope that systems will be revised and implemented in various countries in the future so that appropriate support will be provided to those who need assistance. On the other hand, the daily evolution of technology is astonishing, and techniques using robots are advancing rapidly. Because this is a case report, it is difficult to generalize the progress of this patient to others with this condition. However, we hope to increase the number of positive case reports and data such as those presented here, which will lead to further development.

## Data Availability

All data generated or analyzed during this study are included in this published article.

## Abbreviations

SCI: Spinal cord injury
ADL: Activities of daily living
CSCI: Cervical spinal cord injury
QOL: Quality of life
FIM: Functional Independence measure
ASIA: American Spinal Injury Association
CMV: Controlled mechanical ventilation
SIMV: Synchronized intermittent mandatory ventilation
CPAP: Continuous positive airway pressure
f: Respiratory frequency
PS: Pressure support
PEEP: Positive end-expiratory pressur
PSIF: Posterior spinal instrumentation and fusion
